# “It's your body… so it's just nice to know what they're putting in it” A qualitative study of women’s views and experiences of caesarean section, antibiotic use and infection

**DOI:** 10.1186/s12884-026-09070-9

**Published:** 2026-05-07

**Authors:** N. Pilarski, I. Jones, C. Dunlop, L. Jones, V. Hodgetts-Morton, R. K. Morris, A. Wilson

**Affiliations:** 1 Institute of Applied Health Research, University of Birmingham, Birmingham, United Kingdom; 2 Northern Care Alliance NHS Foundation Trust, Manchester, UK; 3 Institute of Metabolism and Systems Research, University of Birmingham, Birmingham, United Kingdom; 4 Birmingham Women’s and Children’s NHS Foundation Trust, Birmingham, United Kingdom; 5 Liverpool School of Tropical Medicine, Liverpool , United Kingdom

**Keywords:** Caesarean section, Antibiotics, Postpartum infection, Qualitative, Surgical site infection

## Abstract

**Background:**

Over 30% of UK babies are born by caesarean section (CS) and infection after CS is common. Women’s experiences of and views on the management of infection after CS is relatively under explored in the qualitative literature. The aim of this study was to explore women’s views and experiences of (1) CS (2) infection after caesarean section including (3) measures taken to prevent infection such as antibiotics, (4) the use of additional antibiotics and infection prevention measures in research, and (5) identifying infection after discharge home.

**Methods:**

A descriptive qualitative study reported in accordance with Consolidated criteria for Reporting Qualitative research (COREQ) guidelines. Recruitment occurred via social media, and data-collected using semi-structured interviews aided by an evidence informed topic guide. Written or recorded verbal informed consent was obtained. A thematic analysis approach was used to code transcripts, interpret themes and inform a conceptual model of the findings.

**Results:**

Thirteen women were interviewed between December 2021 and March 2022. All women included had had a CS within the last two years, and four had an infection after their CS. For the majority of participants this was their first CS (*n* = 10), they were between 30–40 years old (*n* = 10) and they identified as white British (*n* = 10). Participants had experiences of both planned (*n* = 6) and emergency CS (*n* = 7).

Key themes in how women perceived their experience of CS and infection were ‘knowing my body’, information-sharing and interactions with health systems.. Women’s views on preventative antibiotics, identifying infection and participating in research were mapped to these themes. There was willingness to take part in future research of interventions to reduce the risk of infection after CS.

**Conclusions:**

Further work is needed to develop antenatal information sharing and postnatal follow-up pathways to improve experiences. It is important to ensure women’s voices remain central to future infection prevention research.

**Supplementary Information:**

The online version contains supplementary material available at 10.1186/s12884-026-09070-9.

## Background

Caesarean section (CS) is a common intervention in maternity care – more than 30% of UK babies are now born by CS [1]. Infection after CS affects 8–12% of women and is an important cause of maternal morbidity [2–5]. Infection can occur within the wound itself or within the lining of the womb, both of which are considered surgical site infections (SSI) [6]. CS also increases the risk of other infections such as urinary tract and chest infections [2, 7]. There are many steps that can be taken before, during and after a CS that may help prevent infection. Some of these are already well-evidenced and therefore recommended in national guidelines, including chlorhexidine skin preparations and aqueous iodine vaginal preparations [8]. Other measures, such as body hair removal and incise drapes, have not been studied for use in CS but are not recommended generally in surgery [8, 9]. National guidance states that all women undergoing CS whether planned or as an emergency should receive preventative antibiotics prior to the start of surgery, however, which pre-operative antibiotics should be prescribed is not dictated due to local variation in availability and anti-microbial resistances [8, 10].

Infection after CS can cause many problems for women for example women may need to seek advice and assessment from healthcare providers (HCPs) either in the community or in a hospital, as well as receive potential treatment with antibiotics [11, 12]. Women may need to be readmitted to hospital and face the risk of becoming seriously unwell [3]. Existing research often makes reference to the broader consequences of infection, such as issues with bonding and breastfeeding, the potential impact on the well-being of the new mother trying to care for a new baby whilst unwell, and the anxiety of separation from other children and partners [13, 14]. However, often there is little evidence to support these observations from the perspective of healthcare providers and women’s voices are noticeably absent from the discussion. There has been very little research to try to understand women’s experiences or views of infection after they have undergone a CS. There has also been limited research into women’s views on ways of preventing infection after CS and in particular, their views regarding antibiotics at CS in general, or in the context of research. Previous qualitative research has reported that surgical site infections (SSI) after CS can impact general well-being, with feelings of under-preparedness and lack of information having been commonly interpreted themes [15, 16].

## Methods

### Aims

The aim of this study was to explore women’s views and experiences of (1) caesarean section, (2) infection after caesarean section. (3) measures taken to prevent infection such as antibiotics, (4) the use of additional antibiotics and infection prevention measures in research, and (5) identifying infection after discharge home.

### Study design and setting

This was a pragmatic, descriptive qualitative study [17] with researchers and participants based in the United Kingdom. A descriptive approach was used to allow the views and experiences of participants to be reported in their own words [18]. The methods and findings have been described in accordance with COREQ guidance [19] (Supplement 1).

### Study participants

Recruitment to the WOVAN study was publicised via social media platforms (Twitter, Instagram, Facebook). Interested potential participants were invited to complete an online (Qualtrics XM approved by University of Birmingham) eligibility questionnaire including consent for further contact by the research team.

The eligibility criteria were: (1) the participant had undergone a caesarean section within the last 24 months (with or without developing an infection), (2) aged 16 years or over, and (3) able to provide informed consent. Women were not eligible to participate if they were unable to conduct the interview in English due to language and time constraints.

A convenience sample was used where all eligible participants were followed up by email and provided with study documentation and offered an opportunity to ask questions. An interview was scheduled for all potential participants that responded and wished to participate. All participants provided either signed written consent or recorded verbal consent prior to starting the interview. Confidentiality, the right to withdraw, and permission to record the interview were discussed at the start of each interview. The authors took a pragmatic approach, continuing recruitment until the collected data allowed for a detailed and critical analysis, with sufficient evidence to explore the study aims and develop themes [17]. This was an assessment of the information power, and therefore adequacy, of the sample [20]. After 13 interviews it was considered that an adequate sample had been reached, and so data collection was ceased at this point [21].

### Data collection and analysis

Semi-structured interviews were used to enable detailed and responsive exploration of women’s views and experiences in relation to the study aims [22]. Remote interviewing via teleconferencing software was necessitated by regulations related to the COVID-19 pandemic and reflected the views expressed through patient and public engagement (PPIE) in which women reported a preference for remote interviewing primarily due to its convenience. Interviews were informed by the topic guide (Supplement 2) which was developed following discussion within the study team and literature review. The topic guide included prompts to elicit views and experiences in relation to each of the five study aims. Only the individual researcher and the participant were present for each interview. There were no repeat interviews. Interviews lasted 40–90 min. The automated transcript produced by the teleconferencing software was then quality-checked, corrected, and anonymised by the researcher conducting the interview. Written transcripts were not reviewed by the participants.

For analysis, transcripts were uploaded to NVIVO 12 and analysed using qualitative codebook thematic analysis [23]. Transcripts were coded inductively line by line by NP, or AW or IJ. A collaborative approach was then taken whereby three authors (NP, IJ, AW)) grouped the codes into initial categories based on the study aims and interpreted themes. These were then reviewed and refined into the final themes and a conceptual model was developed illustrating how these themes linked and interacted. To ensure the rigor of analysis a sample of transcripts were in coded in duplicate and an iterative approach to the qualitative thematic analysis was taken such that the original themes were developed and reviewed following further exploration within the research team and re-examination of the original data. The codebook and preliminary themes were discussed within the wider study team to contribute to interpretation and development of the conceptual model.

### Patient and Public Involvement and Engagement (PPIE)

Prior to starting the study, the design and recruitment pathways were discussed with 8 women who had recently had a caesarean section in particular with regards to preferences for approach to participate, consent processes and preferences for methods of data collection (interview – face-to-face or remote, focus groups etc.). This took place in the context of preparatory PPIE work for an allied project relating to prevention of SSI after caesarean section. Those involved expressed a preference for contact by email, individual interviews and an option to participate by phone/remotely (although this was subsequently superseded by COVID-19 guidance to only facilitate phone/remote participation.) The themes, results and final narrative were reviewed and developed in conjunction with PPIE representatives from the Dame Hilda Lloyd Network, a women’s health research collaborative.

## Results

**Fig. 1:**
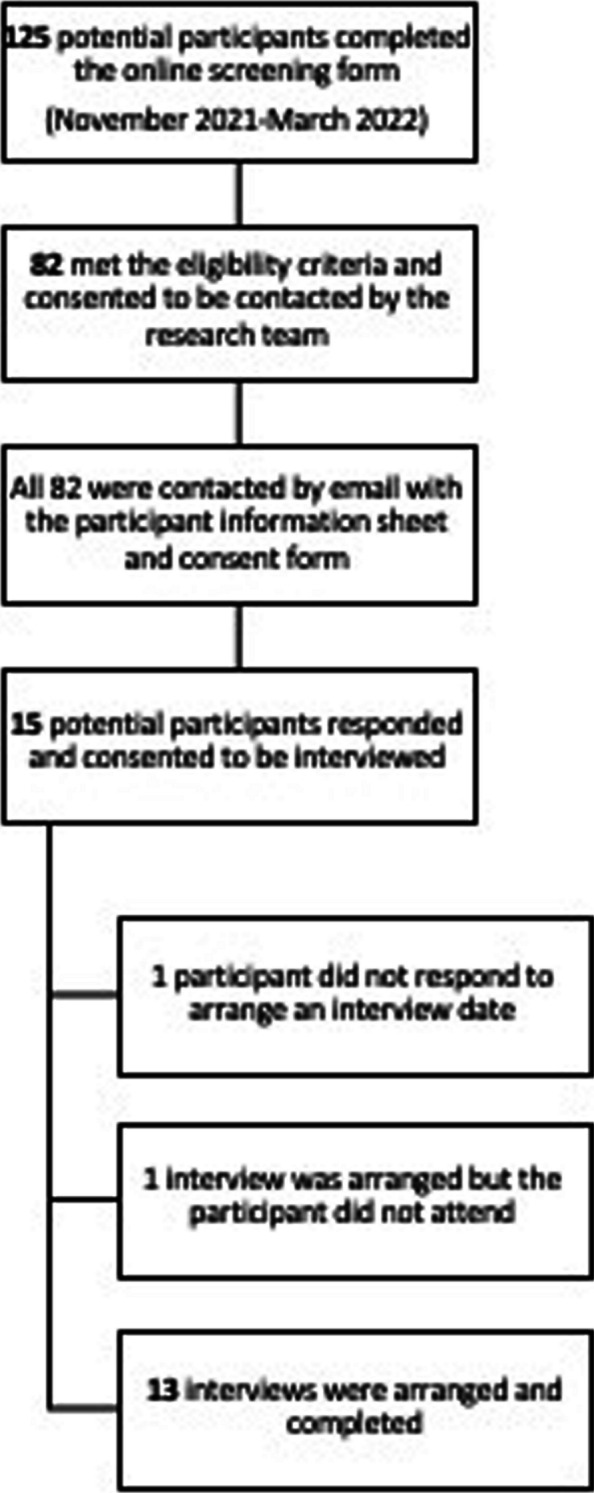
A flowchart of participant engagement and recruitment

A recruitment summary is shown in Fig. 1. Thirteen women were interviewed, four of whom had an infection after their CS. Participant characteristics are presented in Table 1.

**Table 1:** Participant demographics

		*N* = 13
Age	< 25 years	0
	25–34 years	2 (15%)
	35–39 years	9 (69%)
	40–44 years	2 (15%)
	45 + years	0
Ethnicity	Asian or Asian British	1 (8%)
	Black, African, Caribbean or Black British	0
	Mixed or Multiple ethnic groups	2 (15%)
	White	10 (77%)
	Other Ethnic Group	0
	Prefer not to say	0
Infant feeding	Mostly Breastfeeding	6 (46%)
	Mostly Bottle Feeding	5 (38%)
	Both	2 (15%)
Total number of CS	1	10 (77%)
	2	3 (23%)
Infection after most recent CS	Yes	4 (31%)
	No	9 (69%)

*CS* caesarean section

### Developing themes and conceptual models

The key aims of this study were to explore women’s experiences of CS in general and related to infection as well as their views on antibiotic use during or after CS. The research team had, prior to data collection, expected that the recovery experience for women with and without infection after CS would be different. However, from an early stage in the analysis, it was clear that there were common themes relating to the recovery process and infection, even severe infection, was only a part of that process and did not necessarily dominate their recovery experience alongside other common factors such as pain.

**Fig. 2:**
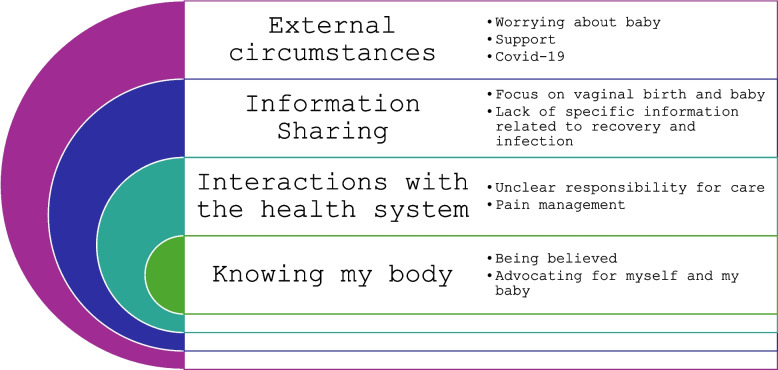
Conceptual model of the key themes influencing experiences after CS reflecting the experiences of women both with and without infection

A conceptual model was developed to demonstrate how individual experience of recovery with and without infection was interpreted and experienced, (Fig. 2). The central key theme is presented in the middle of the figure with influencing themes presented as concentric circles around this. The key themes and contributing sub-themes are presented in Supplement 3 with additional example quotations.

Each of the key themes are discussed below describing how these relate to the the aims of the study – experiences after CS in general, and in relation to infection, as well as women’s views on antibiotic use, identifying infection and future research.

A woman’s interpretation of their own recovery process as a positive or negative experience (or both) were found to be influenced by these key themes We found that a woman’s interpretation of their experience as positive or negative was not strongly influenced by the clinical severity of any complications in their recovery process as may have been expected within a medicalised model of thinking. The research team therefore opted to use the term ‘eventful’ to reflect the experiences of women in that recovery process and how they reported it rather than to describe the recovery process in clinical terms.

The key themes influencing women’s experience of recovery after CS and the role of infection within that were; knowing my body, information-sharing and interacting with healthcare systems. Women’s experiences, and in particular their perception of their CS and postnatal period as positive or negative were influenced by external factors such as the presence or absence of support.

#### Key theme 1: knowing my body

Participants felt that their own understanding of their body was important in seeking advice from her healthcare providers after CS. This was described by one participant as confidence in seeking help.

*“But because I know my body, I think I was more confident in being able to make that judgment myself, as well as to whether I needed to go to a doctor or at which point I needed to go to the doctor or speak to a midwife” (1010, no infection)*

Sub-themes within the theme of ‘knowing my body’ were ‘advocating for myself and my baby’, ‘being believed’.

Particularly for women with infection ‘knowing your body’ was strongly related to women’s feelings of not being believed. Women described knowing that something was wrong because they knew their body but did not feel that healthcare professionals were listening or acting. Being dismissed by her healthcare provider caused one participant to wonder if they did not believe her reported symptoms.

*“I didn’t know if the GP was thinking I was exaggerating or something.” (1026, infection)*

This response from healthcare services had a negative impact on participant’s experiences and was thought to delay access to care. For example, one participant faced a long wait for emergency services.

*“I survived it was all good but for me it was just the frustrations of not being believed by the ambulance service that it was an emergency, because even the hospital said it should have been a level one […] emergency. Also, I was frustrated by the fact that people tried to blame me”* (1027, infection)

Feeling they were not believed or listened to also impacted women after their CS outside of just concerns about infection. When another participant experienced a healthcare provider error, she believed that this was caused by staff not listening to her concerns about her own body.

*“That whole mess was a case of not being listened to.” (1002, no infection)*

Identifying infection was also reflected in women’s perceptions of knowing their own body and how they perceived that in relation to their risk of infection.

“*I'm a bigger girl anyway, I've got- and always have been- I've got quite an overhang on my tummy” (1010, infection)*

With regards to views on participating in future research to prevent infection including antibiotic use women’s perceptions of their own personal risk and a sense of protecting themselves was associated with a willingness to participate for some women. One participant was unsure about participating in research but thought she would agree to a trial of an additional antibiotic, due to her previous experience of infection.

*“Interviewer: Why do you think you would have said yes despite being a bit apprehensive? Participant: Because I think uh… infection is very scary for me” (1026, infection)*

Understanding the risks associated with taking additional antibiotics and how that may impact the women themselves or their baby was represented in the sub-theme of advocating for yourself or you baby.

Participants raised concerns about antibiotic stewardship, adverse effects for themselves and their baby, and whether they would be able to provide genuine informed consent given the timing of antibiotic administration.

One participant recognised the importance of antibiotics in managing infection but wanted to understand the need for an additional antibiotic due to her concern about contributing to antibiotic resistance as the “*the efficacy is vanishing in the long term (1026, infection)*. Three participants were concerned about the effect of antibiotics on the gut microbiome, with two concerned about whether this would also impact their baby.

*“Just to try and minimize the impact of the antibiotics on me and on my baby, you know I want to make sure that she gets a good, healthy flora and I know the antibiotics can impact on the composition of my microbiome and I wouldn't want that to affect hers.” (1056, no infection)*

The potential impact on their baby of either routine or antibiotics in the context of research was shared by other participants. Concerns were raised about their overall well-being and impact of antibiotics on breastfeeding.

*“I guess now and because I said much difficulty with milk production afterwards I would ask that question will that affect milk”. (1004, no infection)*

Informed consent to antibiotic use (within and outside of) research was linked to both knowing your body and specifically the subtheme of advocating for oneself or baby but was also important to the theme of information sharing. The factors reported as influencing women’s decision making regarding participating in research are presented in Table 2 and mapped to the key themes of knowing your body, information sharing and interacting with health systems. Mapping these factors to the key themes demonstrates the dominance of advocating for the safety or added health benefits to oneself or your baby in women’s decision making.

**Table 2:** Factors Affecting Willingness to Participate in Research

Baby (ADVOCATING FOR BABY – a subtheme of knowing your body)	*“If It had been said that it wasn't particularly compatible with breastfeeding, or it could make the baby ill. That would be have been, definitely for me to have it, I think, at that point, I probably would have said no, or seriously considered saying no” 1020* *“i'm assuming you know, in terms of passing through the placenta if everything's okay for baby, that it doesn't make them drowsy or doesn't have any effect on them… and just to make sure can I breastfeed” 1031* *“If everything's okay baby that they… doesn't make them drowsy, doesn't have any effect on them” (1031)*
Recognised treatments (INFORMATION-SHARING)	*“you've got the reassurance of this is tried and tested,” 1029*
Added benefit	*“like if it was less than the original that I was going to get, then no. But if it was same or more than, then yes that's fine” 1030 (INFORMATION-SHARING)* *“Interviewer: Why do you think you would have said yes despite being a bit apprehensive? Participant: Because I think uh… infection is very scary for me” (1026) (KNOWING YOUR BODY)*
Antibiotic resistance (ADVOCATING FOR YOURSELF/BABY)	*“as I know that there's no unnecessary harm going to be done. I mean, I don't know the major effects of antibiotics and things but… yeah so long as I don't [laughs] you don’t get a resistance to antibiotics and there's no major effects on me or, or, or the baby I think yeah”*
Timing (ADVOCATING FOR YOURSELF/BABY)	*“But being an emergency situation, I might have just… been, maybe have been dismissive of it because of being worried about just, just the actual Caesarean. But… so it's hard to say, but… in hindsight I don’t think I would have minded” 1058*
Setting (ADVOCATING FOR YOURSELF/BABY)	*“If they are antibiotics are given, administered when I'm still in hospital where, if anything did go wrong somebody could effectively be right there, then that's fine. I might have been a bit more cautious if it was a case of ‘it's been 24 h, you can go home now and just start taking these” (1010)*

#### Key theme 2: interacting with health systems

The theme of interacting with health systems included communicating with healthcare professionals directly as well as navigating the healthcare system and it’s different pathways. Important sub-themes were the way interactions instilled or undermined women’s confidence in their care, understanding who was responsible for their care, and pain management.

Many women found interactions with health systems confusing frustrating or distressing after their CS. This occurred in multiple situations but particularly in seeking diagnosis, reassurance or treatment for infection after CS as well as in seeking adequate pain relief either both in the context of infection and generally after CS.

Some participants reported a lack of clarity over the responsibilities of different healthcare professionals. For example, one participant felt she received insufficient information about care after CS, but did not know who should have initiated this conversation.

*“I felt like wasn't given much information and I don't know the extent to which midwives are or aren't qualified in terms of surgical aftercare. I genuinely have no idea whether that's part of midwife training or not, but I just think that definitely some sort of debrief while you're in the hospital” (1020, infection)*

This lack of clarity was worsened for one participant when she received conflicting advice as to whom to contact with her concerns about infection.

*“sometimes it's like GP ‘oh you talk to health visitor’ and the health visitor is like ‘no you should talk to the GP’ and it’s like bowling balls from one side to the other.” (1026, infection)*

One participant described the importance to her of healthcare provider follow-up to examine the wound to identify signs of infection.

*‘’Cause I wasn't really sure what, you know, what was a sign of infection. So I would have worried, yeah, without their reassurance. (1058)*

For another participant conflicting views by different healthcare professionals of how the wound was healing and whether there had been an infection was a source of concern.

*“But when I saw a doctor at six weeks nothing really happened, he said, everything was fine when I saw a doctor at nine weeks, he said he thought i'd had an infection and that was why the scar hadn’t healed very well on one side.” (1020 no infection)*

Being in pain was a common narrative in women’s descriptions of their recovery process after CS, both for women with infection in the absence of infection.

*“And then I went back home, they changed their antibiotics and I waited two days, phoned again 111 and then ended up in the A&E and I was just in such pain.” 1026 infection*

Some women, both with and without infection, reported struggling to access adequate pain relief within the health system.

*“The only issues I had post-operatively were them not giving me my painkillers on time which obviously wasn't very good” (1002, no infection)*

One participant felt this reflected a culture rather than a failing on the part of individuals and questioned whether gender or societal expectations played a role in this.

*“And, looking back, I think, God I just had that big surgery and there I am hobbling about with a couple of paracetamol what people take for a headache it just, it’s a bit like, I don’t think it’s the midwives fault, I think it’s just the culture.” (1079, infection)*

*“I can't help but think if we were all men, or if it was a man having a big surgery, would he just have paracetamol?” (1079 infection)*

#### Key theme 3: information-sharing

Information-sharing between healthcare professionals and women was an important factor in many women’s experiences, in particular when there was perceived to be inadequate information. Antenatal classes were reported to focus predominantly on normal vaginal birth, therefore providing too little information about CS and its post-operative recovery process including the risks of infection or how to identify infection.

*“You're not really informed a lot about the fact you could have a C section, and this is the recovery. Whether it's because in all kinda like antenatal classes, they’re kind of obviously more focused on having a natural birth, because that will be the nicest, the best outcome.” (1031, no infection)*

Similarly, a participant felt that healthcare professional’s focus postnatally was on breastfeeding, overlooking recovery and infection.

*“After the baby's out, they jump starkly to breastfeeding and that's the… that seems to be their only concern, breastfeeding and yeah, breastfeeding and that stuff. But no, no, no, not the recovery part or the risk of infection.” (1026, infection)*

Information-sharing (or the lack thereof) about recovery after CS and in particular post-operative pain was a common source of frustration or distress for participating women.

*“I was, you know they say oh you'll be back up and walking and yeah you're walking but like you're barely walking” (1056 no infection)*

Women with and without infection also reported poor information sharing relating to the giving of routine antibiotics at CS (which is routine practice for all CS in the UK). Some women recalled having a discussion regarding the use of antibiotics at CS to prevent infection or being given antibiotics, for others this was not something they recalled happening at all or only became aware of a later stage.

*“I'm fairly sure that I remember being told that they were going to give me IV antibiotics during the time around the procedure, but I don't think I had any after that?” (1002, no infection)*

For some participants it was important to be told in advance, one participant described having faith in the medical team but still wishing to be kept informed.

*“I mean, honestly, I have faith in the medical staff and if they think that that's what's necessary or that will help fight anything, then I'm happy with that. Yeah, I don't have any sort of major objections to it. It would have just been nice to have known that that's what they were doing [laughs]”. (1030, no infection)*

This was also the case for information-sharing about infection-prevention research. Participants all stated that they would be willing to participate in research, although most had additional questions or concerns that they would want to address before giving consent to a specific research study.

*“I would ask, uh, if it's broadly, you know, used? If it has been tested? So… something like that. (1026, infection)”*

As well as information-sharing by healthcare professionals or in structured contexts such as antenatal classes the role of peers in information-sharing also influenced women’s decision making. One participant felt although participating in research involving additional antibiotics did not worry her, she described how her peers discussed their concerns about the long-term impacts of antibiotics on their babies.

*“It’s like the other mums they've been talking like, oh, maybe the baby has this or that because they gave me antibiotics.” (1026, infection)*

Informed consent in the context of participating in infection prevention research was a concern for some women. Participants expressed concerns that, due to the timing of the antibiotic administration, they would not receive all the information they would like before making the decision to participate or not. One participant felt she would decide to participate regardless of this.

*“I would probably make the decision fine I'm just gonna have the antibiotics, but I wouldn't feel like it was like well informed.” (1060, no infection)*

However, another participant felt that even if presented with sufficient information, the timing would not facilitate her making a considered decision.

*“And probably have a little bit of time to weigh that up, to not be presented with it, you know, as you’re sat there on the operating table, but to have a little bit of time to think it through”. (1029, no infection)*

In terms of information-sharing and identifying infection several participants did not feel they would have been able to identify an infection themselves. One participant suggested that being provided with take-home information on identifying infection would have been beneficial but also acknowledged the risk of information overload when first leaving hospital with a newborn.

*Perhaps if it was your first Caesarean maybe some kind of checklist might have been helpful, or it might just be lost in the kind of new baby stuff. (1029)*

The recommendations of participating women for improving follow-up after CS are summarised in Table 3 and the topic areas mapped to the key themes of information-sharing and interacting with health systems.

**Table 3:** Women’s recommendations for improving follow up after CS

Information about CS and recovery (INFORMATION-SHARING)	*“Maybe like a description of what layers they cut through or explaining it. How your wound feels” 1079* *“just reassurance that it might hurt A lot.” 1079*
Breastfeeding (INFORMATION-SHARING)	*“I knew breastfeeding positions from my job, about the best ones to do when you've had a section but for other women, you know there's certain positions which are nicer on the belly.” 1079*
Access to Physiotherapy (HEALTH SYSTEMS)	*“I think if anything I think the other thing that actually has just come to mind that is i'm just astounded by the fact that there's no physio,” 1020*
	*“I was fortunate in that I could go to a physio who specialized in after for women's care.” 1004*
Wound Checks (HEALTH SYSTEMS)	*“with our GP and I asked him, I asked him to check my incision he wasn't he wasn't going to, and I was like just check and make sure it's all okay” 1056*

#### External circumstances

Factors that influenced how women perceived theirrecovery after CS included; support, dependents, role of a partner, worrying about baby. The presence of a support person such as a partner facilitated a positive experience for participants.

*“Okay, during that time, obviously, that was really good, I had a lot of support” (1060, no infection)*

A lack of support was associated with a negative experience for participants. Due to the timeframe of this study, COVID-19 restrictions were in place when most participants were accessing maternity services this impacted on support during birth and the recovery process both for women with and without infection.

Three participants described how their partner performed tasks for them during their recovery. One participant’s partner needed to take additional leave from work to care for the baby while she was experiencing complications related to infection and a repeat hospital admission.

*“My husband had to have a sabbatical because I was totally incapacitated.” (1026, infection)*

COVID-19 restrictions were associated with several participants feeling lonely or isolated in the postnatal period.

*“I was alone always” (1026 infection)*

One participant reported how being separated from her baby affected her experience after CS.

*“My daughter was on the neonatal ward I was just like absolutely desperate to see her”. (1060, no infection)*

However this was not universal that this was a source of distress. One participant experienced complications that required a premature Caesarean section, resulting in her baby requiring additional care after delivery. She did not feel this negatively impacted her experience, in comparison to the *“distress”* (1031, no infection) she felt prior to delivery.

*“He did have to go to the neonatal unit, but it had just been so stressful up to that point so yeah I was quite relieved I made that call”. (1031, no infection)*

## Discussion

This study offers insights into women's experiences of recovery after CS, highlighting the importance of considering the holistic nature of the recovery process. The findings indicate that the key themes influencing women's recovery experiences, included ‘knowing my body’, interactions with health systems, information sharing and external circumstances. The presence and severity of infection, and associated complications with recovery, did not strongly influence a woman's interpretation of their recovery experience. Our findings suggest that measures taken to prevent and identify infection should focus on these shared factors, rather than differentiating by infection status. This challenges a purely medicalised model of thinking, suggesting that subjective factors, such as women’s views about their bodies, pain, concerns for the baby, and support, play pivotal roles in shaping women's perceptions.

When asked about participation in antibiotic research, women reported wanting to contribute to research with a desire to improve the care and experience of women. However, concerns were raised about antibiotic stewardship, adverse effects for themselves and their baby, and whether the timing of antibiotic administration facilitated informed consent.

The findings also emphasise the importance of follow-up to identify potential infections and alleviate women's concerns. Enhanced information-sharing practices specific to CS, both antenatally and postnatally, should be implemented to better prepare women for CS and the subsequent recovery process. Education around the signs of infection post caesarean section is a highlighted gap in these findings, and is recommended for all women undergoing CS by FIGO [24]. Understanding and addressing subjective aspects such as support are crucial for providing comprehensive care and support to women during the recovery process. This will impact their understanding of postnatal infection and health seeking behaviours.

### Existing research

Effective and tailored communication has been highlighted by The World Health Organization (WHO) as a key recommendation for promoting a positive childbirth experience through prioritising respectful maternity care [25, 26]. Respectful maternity care is defined by the WHO as “care organised for and provided to all women in a manner that maintains their dignity, privacy and confidentiality, ensures freedom from harm and mistreatment, and enables informed choice and continuous support during labour and birth” [27]. The WHO state that with the implementation of such practices, healthcare providers can support women in achieving their desired physical, emotional, and psychological birth outcomes for themselves, their babies, and their families [25].

Studies exploring experiences of CS recovery emphasise the need to improve pre-discharge advice and better support for women during the recovery period to improve maternal outcomes after CS. Weckesser et al. [16] stated that participants reported receiving an inadequate amount of information about CS recovery and infection prevention. Kealey et al. [28] highlighted the challenges faced during the recovery period, including physical difficulties encountered included pain, reduced mobility, and wound issues. Similarly, to our study Kealey et al. [28] found that the advice given to women prior to discharge conflicted with their role as new mothers and caregivers, often leading to non-compliance with the recommendations of rest and recovery.

Høgh-Poulsen et al. [29] studied pregnant women’s views on the timing of prophylactic antibiotics during caesarean section. They found that most women preferred to be informed of the use and the timing of prophylactic antibiotics and any associated consequences for trans-placental exposure to the infant. Despite this, similarly to our findings, Weckesser et al. reported that they would participate in research for infection prevention for altruistic reasons [16], although the appropriate timing of seeking informed consent remains in question [5, 30, 31].

### Strengths and limitations

This study benefits from a descriptive qualitative methodology which facilitated the exploration of women’s birth experiences and their views on the use of antibiotics at CS. The analysis was strengthened by the use of researcher triangulation in coding interviews and the development and refinement of the themes and conceptual model.

The results of this study may be subject to recall bias since data were collected retrospectively. To reduce this limitation, participants were only recruited if they had undergone CS within 24 months prior to their interview. Only those who had experienced a CS were included in this study, which may limit transferability of the results to women who have not had a previous CS or for pregnant women for whom it will be their first delivery. There was not parity in the numbers of women participating with and without infection, and while the reported experiences reflected common themes as described in the results, additional insights may have been offered by including more women with infection (all women eligible were invited to participate in interviews).

Participants were asked to discuss their views on their maternity care and birth experience with researchers who are healthcare professionals. Therefore, responses may have been subject to social acceptability bias. To reduce this bias, the use of semi-structured interviews allowed the researchers to establish rapport with the participants [32]. Interviews were conducted individually and participants were assured of anonymity to further facilitate candid responses.

The social context in which this study took place was following the initial wave of the COVID-19 global pandemic but in a timeframe in which restrictions were still in place and many of the women participating will have had significant changes to their antenatal and postnatal care was delivered.

## Conclusion

This study provides insight into the factors which influence women’s experience of recovery following CS both with and without infection, as well as exploring views on infection prevention including research, and identifying infection after CS. The key themes developed were; knowing my body, interactions with health systems and information sharing. External circumstances also influenced women’s experiences. Participants were motivated to participate in antibiotic research by a desire to improve medical care and previous experiences of infection. However, concerns were voiced about antibiotic stewardship, adverse effects of medications and whether the timing of antibiotic administration facilitates informed consent. Further work is needed to promote antenatal information-sharing about infection after CS. Women’s autonomy must be respected and prioritised. This includes ensuring they are involved in the decision-making process to improve recovery experiences. It is important that women’s voices remain central to future infection prevention research so that acceptability of interventions can be assessed.

## Supplementary Information

Supplementary Material 1.

Supplementary Material 2.

Supplementary Material 3.

## Data Availability

No datasets were generated or analysed during the current study.

## Abbreviations

WOVAN: Women’s views of antibiotics at Caesarean Section
CS: Caesarean section
COREQ: COnsolidated criteria for REporting Qualitative research
HCP: Healthcare professional
SSI: Surgical site infection
UoB: University of Birmingham
PPIE: Patient and Public Involvement and Engagement
WHO: World Health Organisation
