# Supplementary material for: “It's your body… so it's just nice to know what they're putting in it” A qualitative study of women’s views and experiences of caesarean section, antibiotic use and infection
Source: BMC Pregnancy Childbirth. 2026 May 7;26:694. doi: 10.1186/s12884-026-09070-9 (PMC13321921; doi:10.1186/s12884-026-09070-9)
Supplement: Supplementary file 2 — Supplementary Material 2. [file 12884_2026_9070_MOESM2_ESM.docx]

Women’s views on antibiotics at caesarean section: A study to explore women’s views on receiving antibiotics at caesarean section

(WOVAN)

**Interviews – Topic Guide**

Interviews will be semi structured. The qualitative researcher will ask open questions followed by further detailed questions where required in order to cover the following topics as appropriate and relevant to the woman participating.

The qualitative researcher and woman participating will explore:

- Experience of caesarean section (CS)
- Knowledge and awareness around antibiotics associated with CS
- Views around additional doses of antibiotics at CS
- Willingness to participate in future maternity trials
- Views on ways to help identify infection after going home from hospital
