# Supplementary material for: “It's your body… so it's just nice to know what they're putting in it” A qualitative study of women’s views and experiences of caesarean section, antibiotic use and infection
Source: BMC Pregnancy Childbirth. 2026 May 7;26:694. doi: 10.1186/s12884-026-09070-9 (PMC13321921; doi:10.1186/s12884-026-09070-9)
Supplement: Supplementary file 3 — Supplementary Material 3. [file 12884_2026_9070_MOESM3_ESM.docx]

| **Supplement 3: Summary table of key themes and sub-themes with examples** | |
| --- | --- |
| **Key Theme 1: Knowing my body** | |
| Knowing my Body | *“with all that stuff that went wrong I didn't trust my body, like as a dancer you kind of know your body and its limits. I’m always in control of my body, you know if I want to do a turn I do to if i'm off balance I know why. From that first birth experience I don't trust my body” 1027*  *“It's your body isn't it so it's just nice to know what they're putting in it” 1079* |
| Being Believed | *“I survived it was all good but for me it was just the frustrations of not being believed” 1027* |
| Identifying infection | *“And obviously there was a- where it was being cleaned, there was a slight odour to it as well. But I think, because… obviously I know my body, and I know that it was in a, what's, I don't really know how to say it… in an area that is quite prone to lots of moisture… I purposely kept a closer eye on it…” 1010* |
| Recovery | *“they come in and they say oh don't do any exercise for so long, and all the rest of it, and i'd read various books from other people who were athletic and everybody was saying, listen to your body, because the advice that they give everybody is kind of for the average person.” 1002 no infection* |
| Being active | *“Much, much more challenging than I expected i'm a really active person… realizing that I couldn't get myself out of that position was really challenging.” 1020* |
| **Key Theme 2: Interactions with health systems** | |
| Pain | *“And, looking back, I think, God I just had that big surgery and there I am hobbling about with a couple of paracetamol what people take for a headache it just, it’s a bit like, I don’t think it’s the midwives fault, I think it’s just the culture.” 1079*  *“And then they, they were like oh yeah lunch is served but you’re going to have to go get it yourself, oh that is just cruel! [laughs] I've just had major surgery, what are you doing to me!” 1030* |
| Choice | *“they kind of said, well, you can either try a breech vaginal birth or go for a C-section, so I jumped at the chance for a C-section” 1010*  *“So I reluctantly agreed to have a caesarean section… Once I decided I was having it, I was all right. I think that the fact that it was my decision really stressed me out a bit” 1020* |
| Identifying infection | *“There wasn't really much direction I didn't, I felt a bit icky looking at it.”1079* |
| Treating infection | *“my GP said mastitis take this antibiotic and to that extent this didn't make any effect, but I phoned 111 and they were like, no, you should go to A&E and then they send me back home.” 1026 infection* |
| **Key theme 3: Information sharing** | |
| Information sharing | *“I was sent home without any instructions I think they said the midwife will take off your dressing, But there was no real, I don’t think they said when and I had no idea and they gave me this postpartum pack, and this paper information, but it didn't mention section it was all vaginal.” 1079*  *“And then when the anaesthetic wore off, contractions came back. I would like what?! No one told me contractions are still happening after the baby's born, and I thought this is over and then this was terrible…” (1026, infection)* |
| Before birth | *“you're not really informed a lot about the fact, you could have a C section, and this is the recovery, whether it's because In all kinda like antenatal classes, they kind of obviously more focused on having a natural birth” 1031* |
| After birth | *“all of that was good and going up to the recovery was fine, but the Ward was awful.” 1020* |
| When things go wrong | *“Everybody was like, no, you have the warm water blah blah blah … And then I went back home, they changed their antibiotics and I waited two days, phoned again 111 and then ended up in the A&E and I was just in such pain.” 1026* |
| Wound care | *“partly from my own knowledge and the midwives did sort of tell me things to look out for.” 1010*  *“the midwife who came to my house recommended: yes you're absolutely fine to shower but make sure that you dry, so just do a little, a little towel dab and then, if you can kind of stomach it, lift your stomach up to let the air get to it, which I did.” 1029* |
| Antibiotics | *“At the time, someone said to me, do you want some extra antibiotics I (was) probably like yeah” 1004*  *“I had already had a birth I didn't really want, well a birth I didn't really expect, and then I suddenly realized I was having all these antibiotics” 1079* |
| Research | *“I'd rather be reassured that things are going to be fine than think about potential side effects from antibiotics, I think. I personally wanted that bit of time after the baby to be… so that I can focus on the baby rather than infected.” 1029*  *“Oral antibiotics I feel like I want to know the justification for having them A bit more. But with the the one surrounding the actual operation it it didn't really bother me.” 1002* |
| **External Circumstances** | |
| Worrying about baby | *“I have to say that was way more traumatic than the delivery and more painful and I have to be in the hospital for days without seeing the baby” 1026*  *“I think… it was difficult looking after the baby with the Caesarean.” 1058* |
| Partner / Support | *“And my husband had to get out of bed, walk round, pick up the baby, put him on me, sort of thing.” 1030*  *“We were very much so trying to fight and and I think if I hadn't had my husband there, and he wasn't the type, who is very happy to advocate for somebody and not be worried about upsetting people, I think I would have been in a really bad state, to be honest.” 1002*  *“I felt a bit guilty, I felt this was our first time really together as a family and instead we were just taking it in teams, because it was my only sort of break”. (1079, infection)* |
| Other dependents | *“I couldn't see my other son as well i've never been away from them and then obviously they couldn't bring them in so, though, when, yes it for 10 days I couldn't see him so that was horrible” 1031* |
| COVID-19 | *“so my partner didn't see us since for two days, basically, after which was so hard like it was so tough being totally alone in the hospital with no support for those first couple of days.” 1056*  *“it was a pretty horrific experience, I mean they were all brilliant, but I think it's just because of the visiting” 1031*  *“the main part was the frustration of like having COVID and being like treated like a leper in a way… I just felt like a nuisance to them” (1027 infection)* |
